# Neural correlates of individual variation in two-back working memory and the relationship with fluid intelligence

**DOI:** 10.1038/s41598-021-89433-8

**Published:** 2021-05-11

**Authors:** Guangfei Li, Yu Chen, Thang M. Le, Wuyi Wang, Xiaoying Tang, Chiang-Shan R. Li

**Affiliations:** 1 Department of Biomedical Engineering, School of Life Sciences, Beijing Institute of Technology, Beijing, China; 2 Department of Psychiatry, Yale University School of Medicine, New Haven, CT USA; 3 Department of Neuroscience, Yale University School of Medicine, New Haven, CT USA; 4 Interdepartmental Neuroscience Program, Yale University School of Medicine, New Haven, CT USA; 5 Connecticut Mental Health Center S112, 34 Park Street, New Haven, CT 06519 USA; 6 Beijing Institute of Technology, 715-3 Teaching Building No. 5, 5 South Zhongguancun Road, Haidian District, Beijing, 100081 China

**Keywords:** Working memory, Intelligence

## Abstract

Working memory has been examined extensively using the N-back task. However, less is known about the neural bases underlying individual variation in the accuracy rate (AR) and reaction time (RT) as metrics of N-back performance. Whereas AR indexes the overall performance, RT may more specifically reflect the efficiency in updating target identify. Further, studies have associated fluid intelligence (Gf) with working memory, but the cerebral correlates shared between Gf and N-back performance remain unclear. We addressed these issues using the Human Connectome Project dataset. We quantified the differences in AR (critical success index or CSI) and RT between 2- and 0-backs (CSI_2–0_ and RT_2–0_) and identified the neural correlates of individual variation in CSI_2–0_, RT_2–0_, and Gf, as indexed by the number of correct items scored in the Raven’s Standard Progressive Matrices (RSPM) test. The results showed that CSI_2–0_ and RT_2–0_ were negatively correlated, suggesting that a prolonged response time did not facilitate accuracy. At voxel p < 0.05, FWE-corrected, the pre-supplementary motor area (preSMA), bilateral frontoparietal cortex (biFPC) and right anterior insula (rAI) showed activities in negative correlation with CSI_2–0_ and positive correlation with RT_2–0_. In contrast, a cluster in the dorsal anterior cingulate cortex (dACC) bordering the SMA showed activities in positive correlation with CSI_2–0_ and negative correlation with RT_2–0_. Further, path analyses showed a significant fit of the model dACC → RT_2–0_ → CSI_2–0,_ suggesting a critical role of target switching in determining performance accuracy. Individual variations in RT_2–0_ and Gf were positively correlated, although the effect size was small (f^2^ = 0.0246). RT_2–0_ and Gf shared activities both in positive correlation with the preSMA, biFPC, rAI, and dorsal precuneus. These results together suggest inter-related neural substrates of individual variation in N-back performance and highlight a complex relationship in the neural processes supporting 2-back and RSPM performance.

## Introduction

Working memory, an executive function that supports higher-level cognition (e.g., fluid intelligence), has been extensively examined using the N-back task, often with brain imaging to investigate the underpinning neural processes^1–4^. Typically, blocks with memory load (e.g., 2-back), in which participants are required to track and remember the sequence of stimuli, and blocks of 0-back, in which participants are required to detect a pre-specified target, are inter-mixed^5,6^. As 2-back requires both attentional monitoring and working memory to keep track of the stimuli while 0-back requires only attentional monitoring of the target, a contrast of brain activation during 2-versus 0-back would highlight regional responses specific to working memory. Investigators quantify the accuracy rate (AR) and reaction time (RT) to evaluate individual performance, with a higher AR and shorter RT during correct responses of 2-relative to 0-back indicating superior working memory. However, whereas abundant research has characterized the regional responses to N-back memory, relatively little is known about the neural processes supporting individual difference in AR_2–0_ or RT_2–0_ or whether these neural processes are inter-related. Earlier work showed that activations of bilateral premotor and/or lateral prefrontal cortex (PFC) during 3- vs. 1-back or fixation were associated with higher 3-back accuracy across subjects^7,8^. A more recent study demonstrated that the linear slope of load-related activity (from 1- to 6-back) of the left lateral PFC was associated with individual accuracy in target identification^3^. No studies to our knowledge have examined the neural correlates of RT or addressed whether or how the neural correlates of AR and RT may be inter-related in the N-back task.

Distinguishing the neural mechanism subserving individual variation in AR_2–0_ and RT_2–0_ is of conceptual interest in understanding the psychological constructs of working memory. On one hand, successful performance requires both maintenance of the stimuli in the memory (keeping track of the 1-back stimulus when the 2-back stimulus represents a potential target) and focus switching (updating the target identity with the appearance of each new stimulus)^9^. Thus, a high AR reflects superior capacity in maintenance and updating, whereas a short RT more narrowly reflects the ability in target switching/updating. Individuals with neuropsychiatric conditions typically perform worse compared to healthy controls by showing diminished AR and prolonged RT in the N-back task^10^. On the other hand, with few exceptions (e.g.^11^, the great majority of N-back studies have not purposely constrained the RT by imposing a response window; hence, participants may slow down to optimize AR. In other words, it is possible that participants are slower but more accurate in identifying the target, resulting in a ceiling effect on the AR and rendering the RT of correct responses a more sensitive metric of individual performance^12,13^. Examining the potentially distinct neural underpinnings of the AR and RT (i.e., AR_2–0_ and RT_2–0_) would facilitate research of working memory and how working memory relates to higher-level cognition such as fluid intelligence (Gf).

Working memory is strongly related to Gf. As one of the primary predictors of Gf, working memory contributes to Gf via the process of central executive or cognitive control^14–18^. Moreover, training on working memory appears to improve Gf^19,20^. The close link between working memory and Gf may result from shared underlying neural mechanisms. For instance, bilateral prefrontal and parietal cortical activity accounts for a significant proportion of the shared variance in working memory and Gf^21^. Nevertheless, it remains unclear whether or how individual variation in Gf is reflected in the AR or RT of the N-back task and how regional brain activation inter-links Gf and the performance measures.

The current study aimed to address these issues by using a large data set curated from the Human Connectome Project (*n* = 949). We contrasted brain activities between the 2- and 0-back blocks to control for the effects of attentional monitoring within subjects. We computed a critical success index (CSI) to reflect AR and the RT of correct trials for individual subjects. We performed a whole-brain regression of 2- vs. 0-back each against the block differences (i.e., 2- minus 0-back) in CSI_2–0_ and in RT_2–0_, with age, sex and years of education as covariates, to identify the regional correlates. We also performed a whole-brain linear regression against the PMAT24_A_CR, the number of correct responses in the Raven’s Standard Progressive Matrices, to identify regional responses to Gf. We hypothesized that individual variations in CSI_2–0_ and RT_2–0_ are associated with both distinct and shared regional activities and that some of these regional processes reflect individual variation in Gf.

## Materials and methods

### Data set

We employed the 1200 Subjects Release (S1200) data set, including behavioral and 3 T MR imaging data of 1206 healthy young adults collected from 2012 to 2015, for this study. Of all 1206 subjects, 124 did not participate or participate fully in the N-back task. Further, 133 subjects who had head movements greater than 2 mm in translation or 2 degrees in rotation or for whom the images failed in registration to the template were excluded. As a result, a total of 949 (493 women; 22–37 with mean ± SD = 28.8 ± 3.7 years) were included in the current study. Individuals were without documented history of psychiatric, including developmental (e.g., autism), neurological (e.g., Parkinson’s disease), or medical (e.g., diabetes) disorders known to influence brain function. Twins/non-twins born prior to 34/37 weeks of gestation were excluded. The HCP included smokers, alcohol drinkers and users of illicit substances as long as they did not experience severe symptoms (e.g., inability to stop using; substance abuse despite health consequences) or receive treatment for substance use, so that the data collected of the study reflected the broader populations^22^. We have obtained permission from the HCP to use the Open and Restricted Access data. Participants provided written informed consent and all aspects of the study, including subject recruitment, experimental procedures were conducted according to a protocol in accordance with the Declaration of Helsinki and approved by the Washington University Institutional Review Board (IRB #201204036; title: “Mapping the Human Connectome: Structure, Function and Heritability”).

The Raven’s Standard Progressive Matrices (RSPM) is a 60-item test of abstract reasoning, a nonverbal estimate of fluid intelligence (Gf). All HCP participants were evaluated with the Form A, an abbreviated version of the RSPM with 24 items and 3 bonus items, arranged in order of increasing difficulty^23^. Participants were instructed to complete all items or until they made 5 incorrect responses in a row. The total number of correct responses was coded as PMAT24_A_CR, which we employed as an index of Gf in the current work.

### N-back task

**Figure 1:**
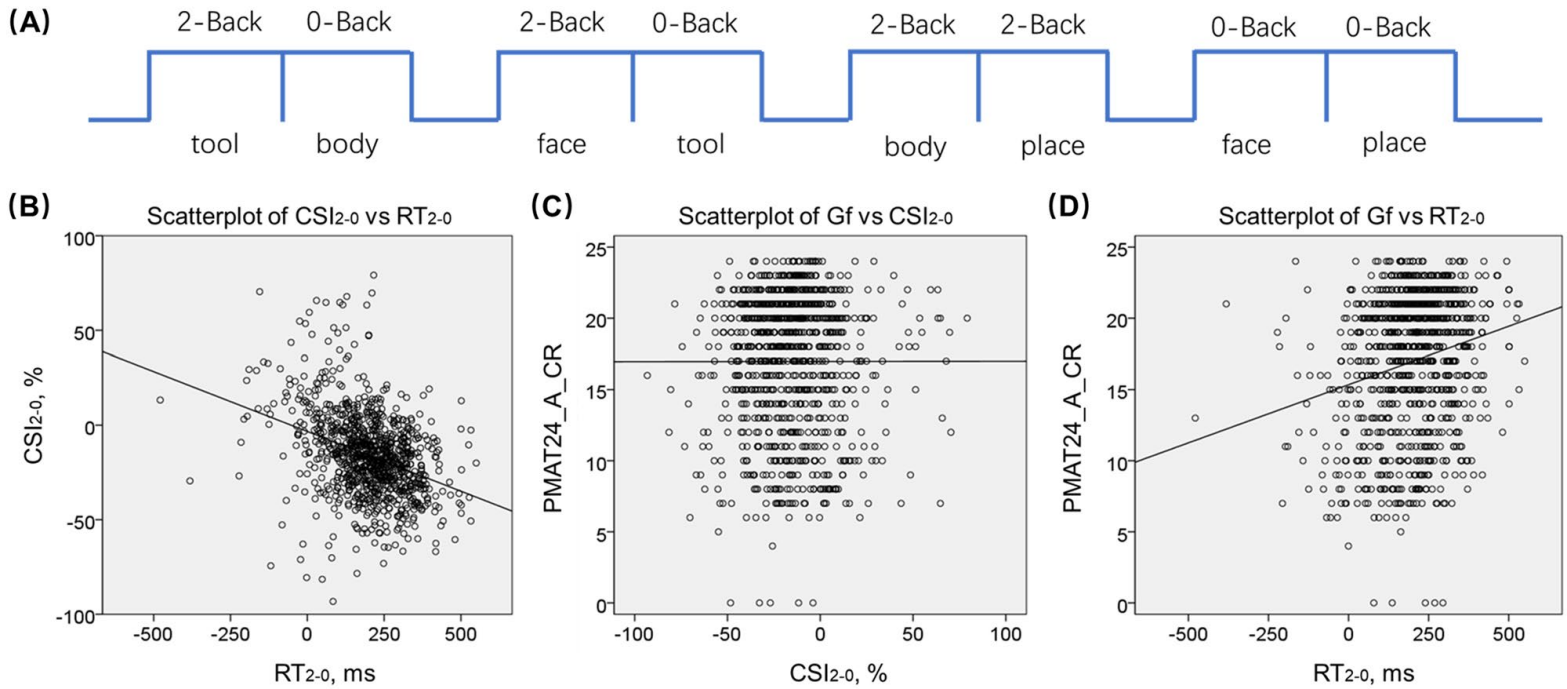
N-back task and the correlation of CSI_2–0_ (%) and RT_2–0_ (ms) with Gf. (**A**) Block sequence of the first run of the N-back task. Linear regression of (**B**) the difference in critical success index (CSI) vs. the difference in reaction time (RT) of correct trials between 2- and 0-back: CSI_2–0_ vs. RT_2–0_ (*r* = -0.350, Cohen’s f^2^ = 0.140, *p* = 1.1588 × 10^–28^); (**C**) Gf, as indexed by PMAT24_A_CR vs. CSI_2–0_ (*r* = 0.006, Cohen’s f^2^ = 3.6 × 10^–5^, *p* = 0.847); and (**D**) PMAT24_A_CR vs. RT_2–0_ (*r* = 0.155, Cohen’s f^2^ = 0.0246, *p* = 0.000002), with age, sex, and years of education as covariates. Each data point represents one subject. B, C and D were generated by SPSS Statistics 22.0 (https://www.ibm.com/support/pages/spss-statistics-220-available-download).

Each subject completed two runs each of eight blocks (four 0- and four 2-back) of the N-back task in a fixed order (first run: 2,0,2,0,2,2,0,0; second run: 2,0,2,0,0,2,0,2). Four categories of stimuli (body part, face, place, tool) were used in individual blocks. The first run was shown in Fig. 1A. In each block a cue was presented for 2.5 s to indicate the current task (0- or 2-back, including target for 0-back) at block start. In 0-back participants were to identify the specified target and in 2-back blocks participants identified the target, a cue that was the same as the one that appeared two time steps back. A “null” block of 15 s was inserted every two blocks in the task. There was a total of 10 trials in each block, of which 2 were targets and 2–3 were non-target lures (i.e., same items in wrong n-back position, either 1-back or 3-back). In each trial, the stimulus was presented for 2 s, followed by an inter-trial interval of 0.5 s. Before undergoing MR scans, participants were engaged in a practice session in a mock scanner to become familiar with the task and acclimated to the environment^22^.

We used the Critical Success Index (CSI) to evaluate N-back performance. A modified estimate of percent accuracy, the CSI was defined as: hits number (correct intentional responses) divided by the sum of hits, false alarms (incorrect intentional responses), and misses (incorrect intentional non-responses)^24,25^. Because correct intentional non-responses (“rejections”) could not be discriminated from correct unintentional non-responses, the CSI was preferred over standard percent accuracy. That is, percent accuracy as computed conventionally resulted in overinflated accuracy estimates, especially for designs with a high percentage of non-response trials, as in the present study (∼ 80% of trials). Thus, the CSI provided a performance measure less biased by the ambiguity of non-response trials^24,25^.

For linear regression between CSI and RT, CSI and Gf, RT and Gf, the effect sizes were quantified by Cohen’s f^2^ = r^2^/(1 − r^2^) and interpreted following the convention: 0.02 ~ small, 0.15 ~ medium, 0.35 ~ large^26^.

### Imaging protocol and data preprocessing

In the HCP imaging protocol^27^, MRI scanning was done using a customized 3 T Siemens Connectome Skyra using a standard 32-channel Siemens receiver head coil and a body transmission coil. T1-weighted high-resolution structural images were acquired using a 3D MPRAGE sequence with 0.7 mm isotropic resolution (FOV = 224 × 224 mm, matrix = 320 × 320, 256 sagittal slices, TR = 2400 ms, TE = 2.14 ms, TI = 1000 ms, FA = 8°) and used to register functional MRI data to a standard brain space. N-back fMRI data were collected using gradient-echo echo-planar imaging (EPI) with 2.0 mm isotropic resolution (FOV = 208 × 180 mm, matrix = 104 × 90, 72 slices, TR = 720 ms, TE = 33.1 ms, FA = 52°, multi-band factor = 8, 405 frames, ~ 4 m and 51.6 s/run).

We followed the same published routines in our earlier studies^28,29^. Imaging data were preprocessed using SPM8. Images of each individual subject were first realigned (motion corrected). A mean functional image volume was constructed for each subject from the realigned image volumes. These mean images were co-registered with the MPRAGE image and then segmented for normalization with affine registration followed by nonlinear transformation. The normalization parameters determined for the structural volume were then applied to the corresponding functional image volumes for each subject. Finally, the images were smoothed with a Gaussian kernel of 4 mm at Full Width at Half Maximum.

### Imaging data modeling and statistics

We modeled the BOLD signals to identify 2-back and 0-back responses. We followed our previous routine in image data modeling^28,29^. A statistical analytical block design was constructed for each individual subject, using a general linear model (GLM) by convolving the canonical hemodynamic response function (HRF) with a boxcar function in SPM. Realignment parameters in all six dimensions were entered in the model as covariates. The GLM estimated the component of variance that could be explained by each of the regressors.

In the first-level analysis, we constructed for each individual subject a statistical contrast “2- minus 0-back” for second-level, group analyses. In group analyses, we conducted a one-sample t test of the contrast “2- minus 0-back” to identify regional responses to working memory. In addition to the T maps, effect size maps were computed using tools available in CAT12 toolbox (http://www.neuro.uni-jena.de/cat/), by approximating Cohen’s d^26^ from the t-statistics using the expression $d=\frac{2t}{\sqrt{df}}$ as employed in^30^. To examine how regional brain activations to working memory varied across subjects in relation to accuracy and RT, we conducted whole-brain multiple regressions each on the contrast (2- minus 0-back) against differences in critical success index (2- minus 0- back; CSI_2–0_) and differences in RT (2- minus 0-back; RT_2–0_) of correct trials only as the regressor, with age, sex and years of education as covariates. We performed another whole-brain multiple regression on the contrast (2- minus 0-back) against PMAT24_A_CR with the same covariates.

We evaluated the results at voxel *p* < 0.05, corrected for family-wise error (FWE) of multiple comparisons, on the basis of Gaussian random field theory, as implemented in SPM. We identified brain regions using the Data Processing & Analysis of Brain Imaging toolbox (DPABI)^31^ and an atlas^32^, if the peak was not identified by the DPABI.

### Path analyses

We employed path analysis to evaluate how CSI_2–0_, RT_2–0_, and the neural correlates (see “Results”) were inter-related. Model fit was assessed with standard fit indices which included the Root Mean Square Estimation of Approximation (RMSEA, < 0.08 for an acceptable fit), Chi-square (χ^2^/df, < 3), Comparative Fit Index (CFI, > 0.9), and Standardized Root Mean Square Residual (SRMR, < 0.06)^33–35^.

## Results

### Behavioral performance and its relationship to fluid intelligence

Subjects averaged at a CSI of 73.9 ± 22.3 (mean ± S.D.) % in 0-back and 58.0 ± 19.6% in 2-back, and a RT (correct trials only) of 790 ± 138 ms in 0-back and 989 ± 137 ms in 2-back. The CSI was significantly lower in 2- than in 0-back (*t* = − 21.695, Cohen’s d = -1.14, *p* = 4.6532 × 10^–85^, paired-sample t test) and the RT was significantly longer in 2- than in 0-back (*t* = 49.217, Cohen’s d = 3.20, *p* = 2.3886 × 10^–263^, paired-sample t test). Across subjects, the differences in CSI between 2- and 0- back, i.e., CSI_2–0_, was negatively correlated with the differences in RT between 2- and 0- back, i.e., RT_2–0_ (*r* = -0.350, Cohen’s f^2^ = 0.140, *p* = 1.1588 × 10^–28^, Pearson regression with age, sex and years of education as covariates, Fig. 1B). That is, higher CSI_2–0_ was associated with smaller RT_2–0_.

Across subjects, the Gf, as indexed by the PMAT24_A_CR, was not correlated with CSI_2–0_ (*r* = 0.006, Cohen’s f^2^ = 3.6 × 10^–5^, *p* = 0.847, Fig. 1C) but positively correlated with RT_2–0_ with a small effect size (*r* = 0.155, Cohen’s f^2^ = 0.0246, *p* = 0.000002, Fig. 1D) in Pearson regression with age, sex and years of education as covariates.

### Regional activations to 2- vs. 0-back

**Figure 2:**
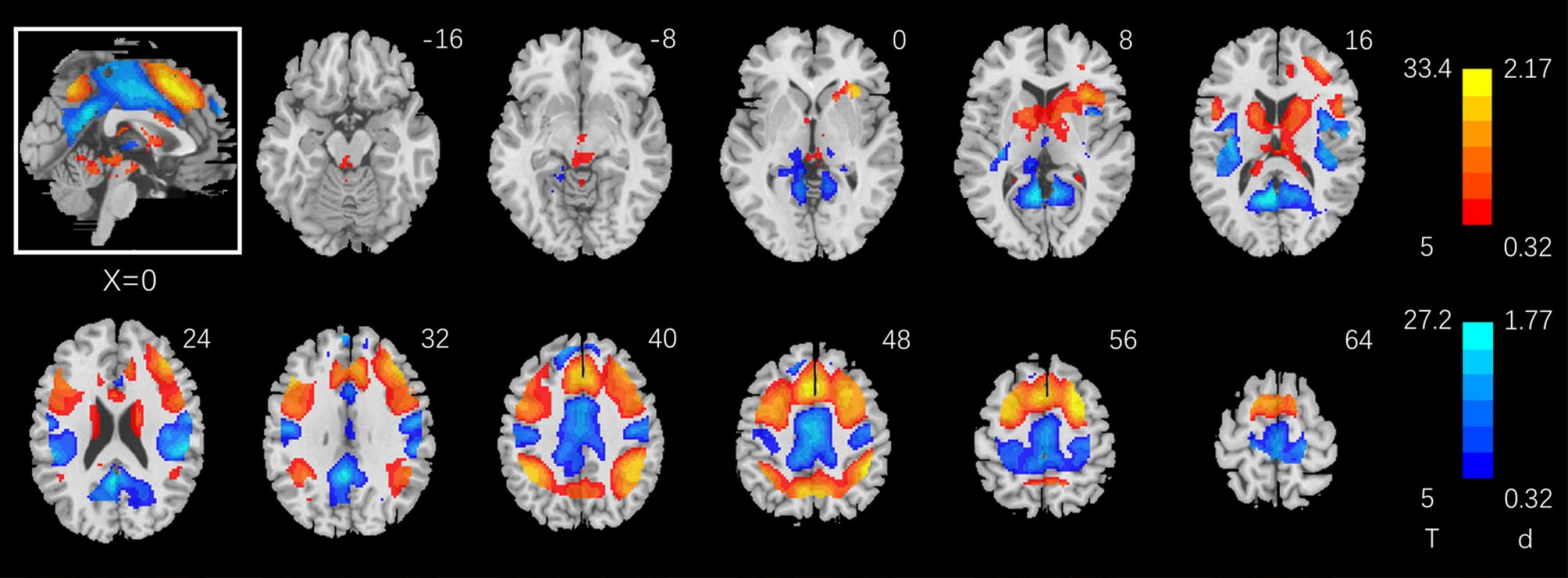
One-sample T-test of the contrast 2- minus 0-back. Voxel *p* < 0.05, FWE corrected. Voxels showing higher activity during 2- vs. 0-back and 0- vs. 2-back are shown in warm and cool colors, respectively. BOLD contrasts are overlaid on a structural image in axial sections from *z* = − 16 to + 64 with 8 mm gaps. Color bars show voxel T values and the corresponding Cohen’s d scores. Neurological orientation: right = right. The inset shows a mid-sagittal section of the clusters. Figures 2, 3, 4, 5A and 7A were generated by DPABI_V4.0_190305 (http://rfmri.org/dpabi).

Figure 2 shows the results of one-sample t test of 2- vs. 0-back in a whole-brain analysis. Two- vs. 0-back involved higher activation in bilateral superior/middle/inferior frontal cortex, bilateral inferior parietal cortex, medial frontal cortex in the area of pre-supplementary motor area (preSMA), extending to an area just anterior to the dorsal anterior cingulate cortex (dACC), bilateral caudate/lentiform nucleus, anterior thalamus, anterior insula, superior parietal lobule, including the dorsal precuneus. Conversely, 0- vs. 2-back involved higher activation in the dACC, middle/posterior cingulate cortex, bilateral somatomotor cortex, paracentral lobule, ventral precuneus, frontopolar cortex, and thalamus in the area of pulvinar and habenula. Many of these brain regions were contiguous to form larger clusters, as summarized in Supplementary Table S1.

### Regional activations to 2- vs. 0-back in correlation with CSI_2–0_ and RT_2–0_

**Figure 3:**
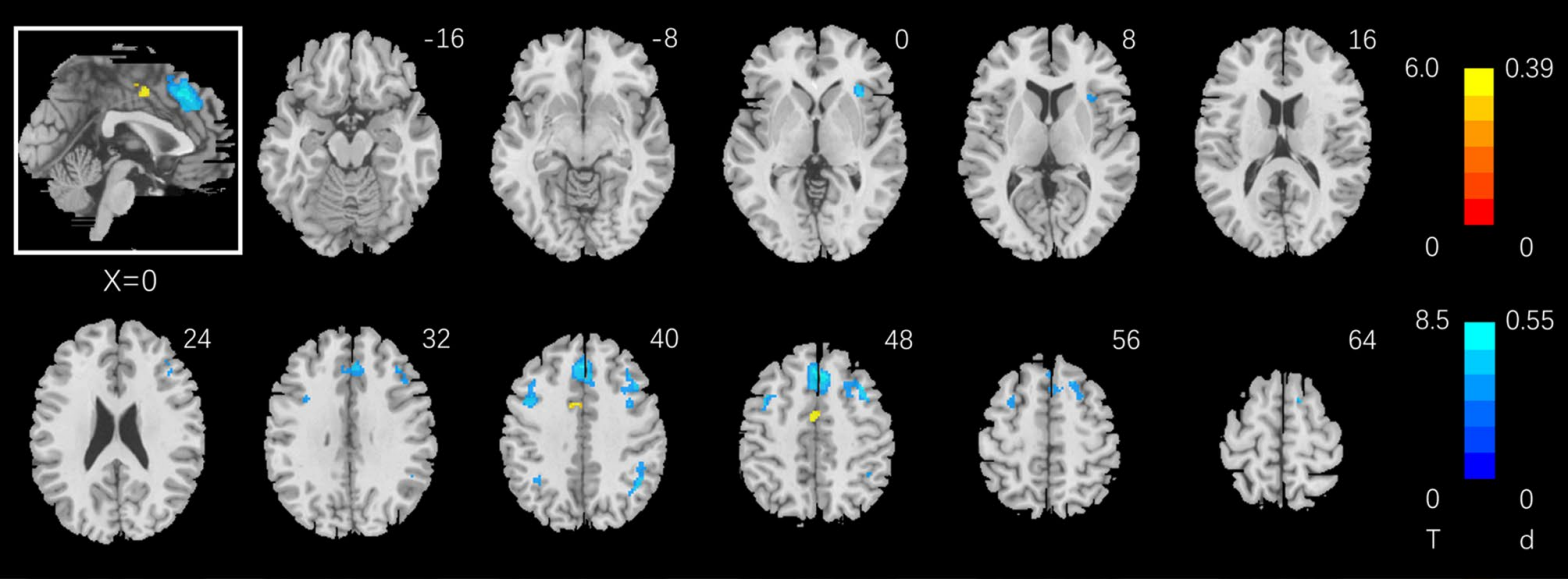
Whole-brain multiple regression against CSI_2–0_ with age, sex, and years of education as covariates. Voxel *p* < 0.05, FWE corrected. Voxels in warm/cool colors show positive/negative correlations. Color bars show voxel T values and the corresponding Cohen’s d scores. The inset shows a mid-sagittal section of the clusters.

**Figure 4:**
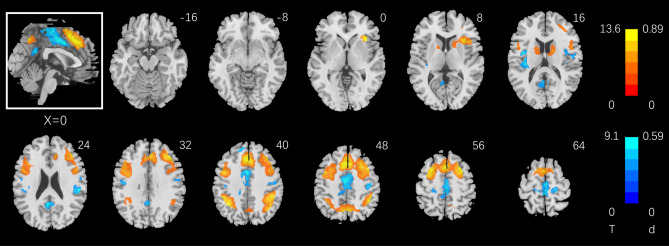
Whole-brain multiple regression against RT_2–0_ (correct trials only) with age, sex, and years of education as covariates. Voxel *p* < 0.05, FWE corrected. Voxels in warm/cool colors show positive/negative correlations. Color bars show voxel T values and the corresponding Cohen’s d scores. The inset shows a mid-sagittal section of the clusters.

Whole-brain linear regression against CSI_2–0_ shows regional activations in Fig. 3 (clusters summarized in Table 1). Briefly, CSI_2–0_ was correlated positively with activation of a small cluster located in the dorsal anterior cingulate cortex (dACC) bordering the supplementary motor area (SMA) and negatively with activation of the preSMA, bilateral frontoparietal cortex (biFPC), and right anterior insula (rAI). Figure 4 shows regional activations to 2- vs. 0-back in correlation with RT_2–0_ (clusters summarized in Table 2). RT_2–0_ was positively correlated with activation in biFPC, preSMA, rAI, caudate head and dorsal precuneus, and negatively correlated with activation of a large cluster extending from the midcingulate cortex to paracentral lobule, ventral precuneus, bilateral primary motor cortex, middle/posterior insula, and right superior temporal sulcus.

**Table 1:** Clusters showing correlation with CSI_2–0_ (Critical Success Index), with age, sex, years of education as covariates.

Region	Cluster size (k)	Peak voxel (T)	Cluster FWE P-value	MNI coordinate (mm)		
				X	Y	Z
**Positive**						
Dorsal ACC*	60	5.95	0.000	− 2	− 2	46
**Negative**						
Frontal_Sup_Medial	1246	− 8.53	0.000	4	26	46
Precentral_L	275	− 7.08	0.000	− 42	8	42
Angular_R	153	− 6.86	0.000	44	− 54	40
Insula_R	111	− 6.40	0.000	34	18	2
Parietal_Inf_L	41	− 5.63	0.000	− 36	− 54	42

*ACC* anterior cingulate cortex, *R* right, *L* left.

**Table 2:** Clusters showing correlation with RT_2–0_ (correct trials only), with age, sex, years of education as covariates.

Region	Cluster size (k)	Peak Voxel (T)	Cluster FWE P-value	MNI coordinate (mm)		
				X	Y	Z
**Positive**						
SMA/preSMA/biFPC/rAI/Caudate_R	7970	13.64	0.000	− 6	16	48
Parietal_Inf_L/Parietal_Inf_R/dPCu	2220	12.60	0.000	− 35	− 54	42
Caudate_L	158	7.69	0.000	− 16	0	20
**Negative**						
SupraMarginal_R	396	− 9.09	0.000	52	− 28	26
Cingulum_Mid	1792	− 8.74	0.000	− 2	− 6	46
Cingulum_Post	422	− 8.39	0.000	− 2	− 52	26
Rolandic_Oper_L	350	− 8.34	0.000	− 38	− 16	20
Frontal_Sup_Medial_L	50	− 7.35	0.000	− 10	48	40
Postcentral_R	240	− 7.09	0.000	38	− 20	46
Postcentral_L	128	− 6.66	0.000	− 44	− 14	34

*R* right, *L* left, *SMA* Supplementary Motor Area, *biFPC* bilateral frontoparietal cortex,* rAI* right anterior insula, *dPCu* dorsal precuneus

**Figure 5:**
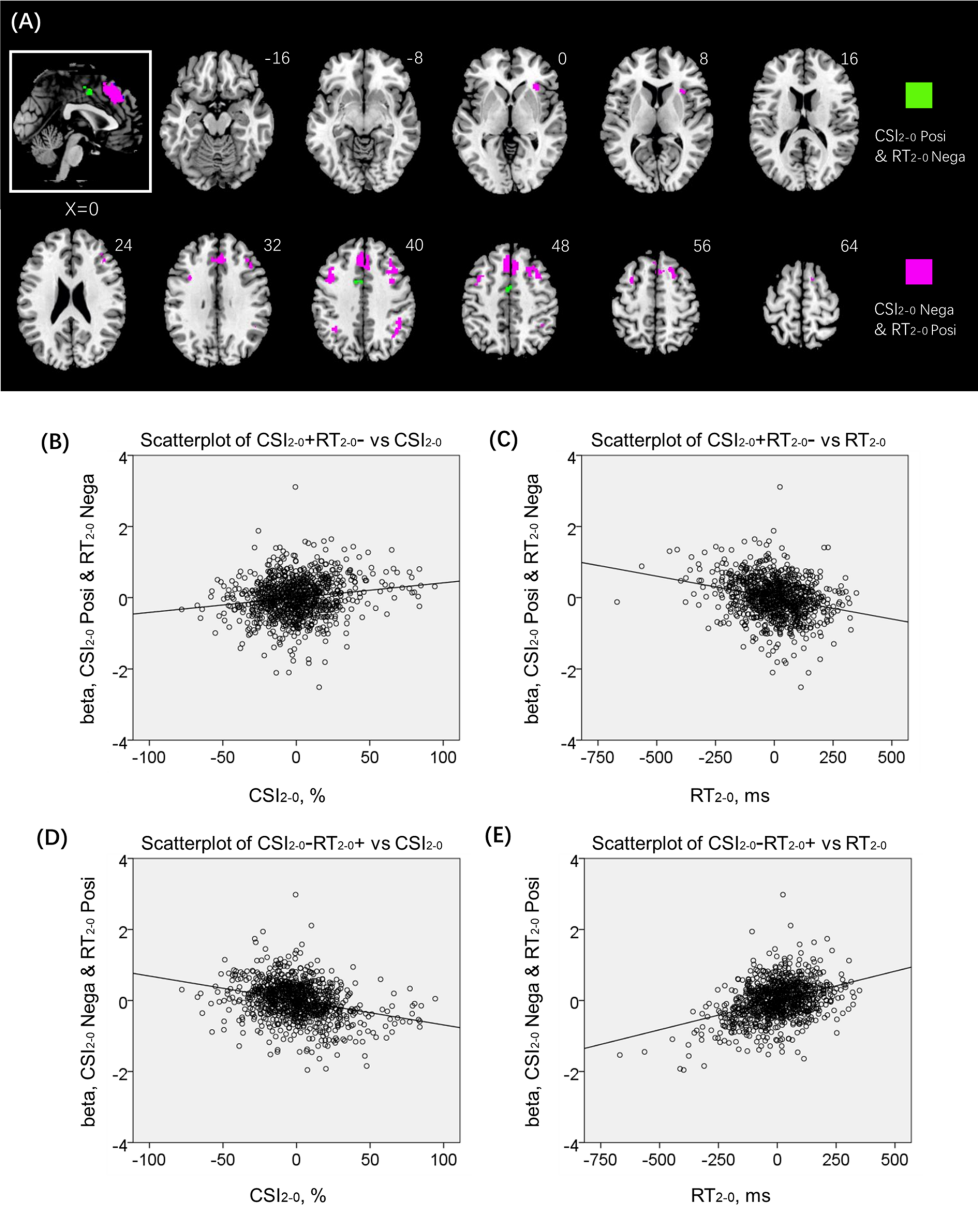
(**A**) A number of clusters showed positive correlation with CSI_2–0_ and negative correlation with RT_2–0_ (CSI + RT −), highlighted in green, or negative correlation with CSI_2–0_ and positive correlation with RT_2–0_ (CSI − RT +), highlighted in magenta. The CSI + RT − cluster comprised solely of the dACC. Linear regression of (**B**) beta estimates of green cluster (CSI + RT −) vs. the difference in CSI between 2- and 0-back: CSI + RT − vs. CSI_2–0_; (**C**) beta estimates of green cluster (CSI + RT −) vs. the difference in RT of correct trials between 2- and 0-back: CSI + RT − vs. RT_2–0_; (**D**) CSI − RT + vs. CSI_2–0_; and (**E**) CSI − RT + vs. RT_2–0_. Note that the scatter plots show the residuals, after age, sex, and years of education were accounted for. (**B**–**E**) were generated by SPSS Statistics 22.0 (https://www.ibm.com/support/pages/spss-statistics-220-available-download).

**Figure 6:**
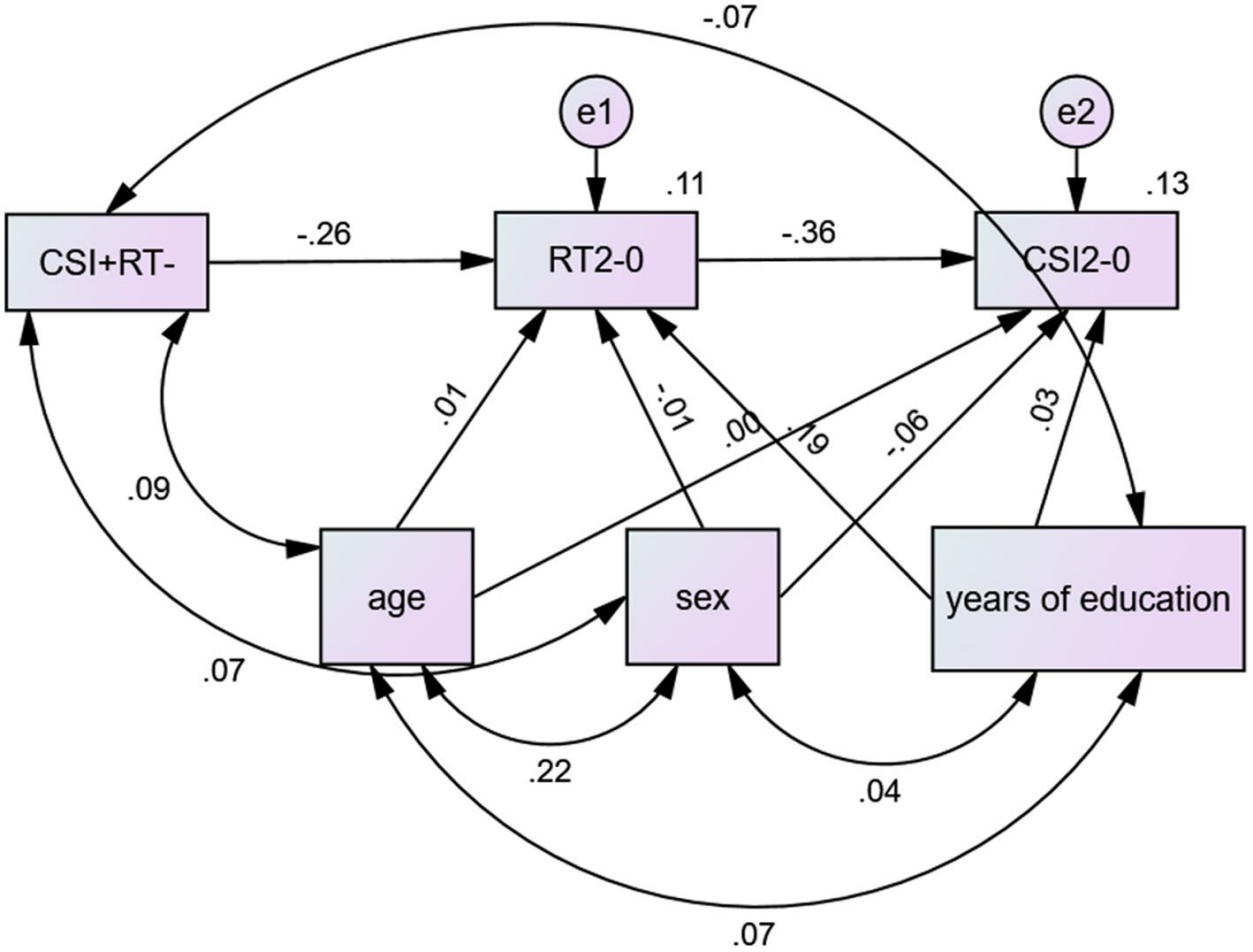
The model of CSI + RT − → RT_2–0_ → CSI_2–0_, which showed the best fit of all 12 models, is shown with path coefficients, covariates and covariance structures. Figure was generated by IBM SPSS Amos 22 (https://www.ibm.com/support/pages/downloading-ibm-spss-amos-22).

A number of clusters showed positive correlation with CSI_2–0_ and negative correlation with RT_2–0_ (CSI + RT −; dACC) or negative correlation with CSI_2–0_ and positive correlation with RT_2–0_ (CSI − RT +; preSMA, biFPC, and rAI) (Fig. 5). These shared regional activities may represent the neural substrates interlinking accuracy and RT in the N-back task. Thus, we performed path analyses to examine the inter-relationship between the shared correlates (CSI + RT − or CSI − RT +), CSI_2–0_, and RT_2–0_. For the sake of completeness, we evaluated all 12 models, although the models with CSI + RT − and CSI − RT + as dependent variables were conceptually unlikely. The results of path analyses showed the model CSI + RT- → RT_2–0_ → CSI_2–0_ with the best fit (Fig. 6), suggesting that the dACC may facilitate target switching during the stimulus stream and target identification accuracy. Supplementary Table S2 shows the statistics of all other models (Supplementary Fig. S2). In contrast, the counterpart CSI − RT +  → RT_2–0_ → CSI_2–0_ or any other models did not show a significant model fit.

### Regional activations to 2- vs. 0-back in correlation with Gf (PMAT24_A_CR)

**Figure 7:**
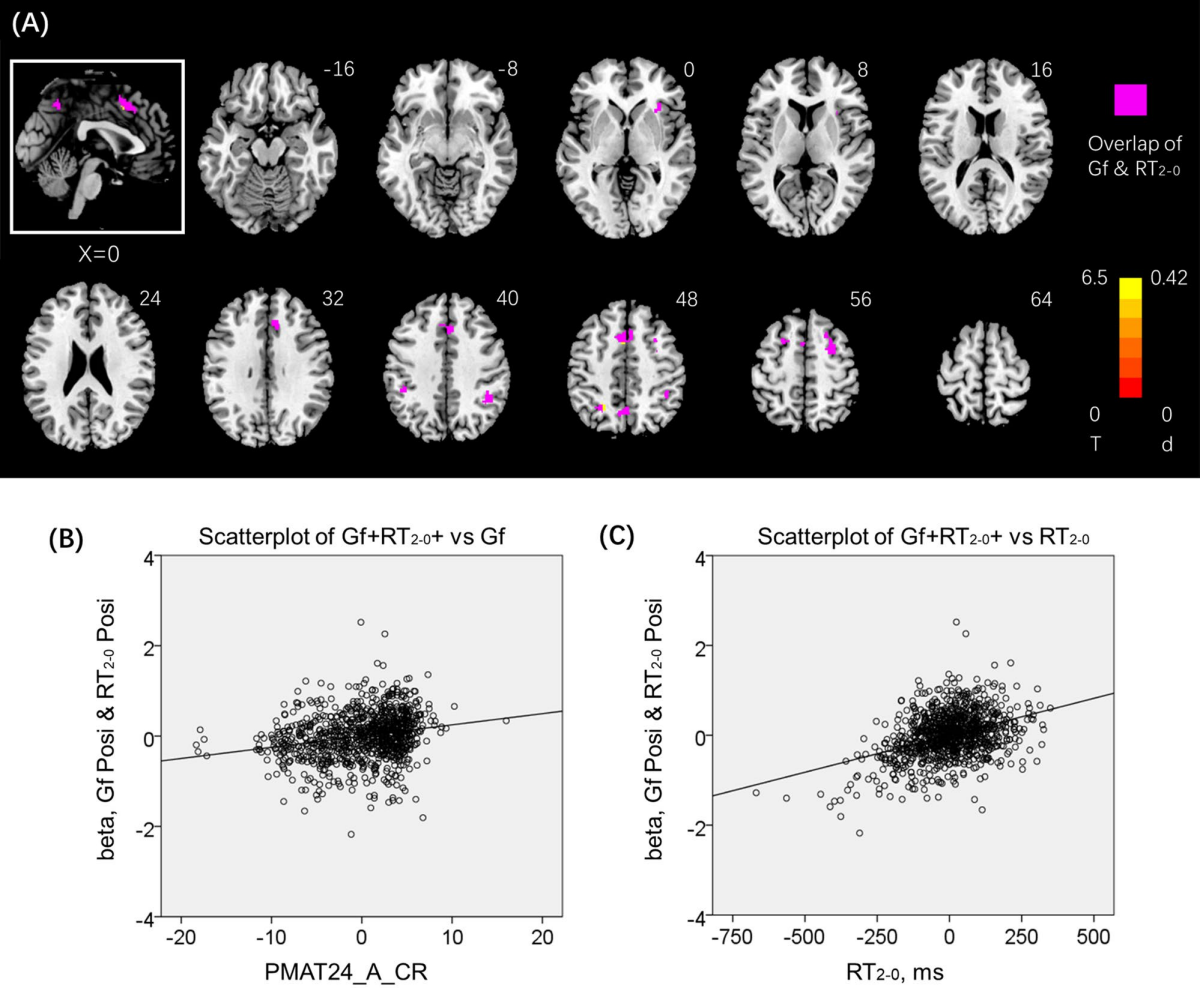
(**A**) Whole-brain multiple regression against PMAT24_A_CR with age, sex, and years of education as covariates. Voxel *p* < 0.05, FWE corrected. The clusters are summarized in Table 3. Clusters in warm color show higher activation during 2- vs. 0-back blocks in positive correlation with PMAT24_A_CR. Color bars show voxel T values and the corresponding Cohen’s d scores. The inset shows a mid-sagittal section of the clusters. The voxels (almost all) with activation in positive correlation with both Gf and RT_2–0_ are highlighted in magenta. Linear regression of (**B**) beta estimates of the magenta clusters (Gf + RT +) vs. Gf (PMAT24_A_CR): Gf + RT + vs. Gf; and **(C)** Gf + RT + vs. RT_2–0_. Note that the scatter plots show the residuals, after age, sex, and years of education were accounted for. B and C were generated by SPSS Statistics 22.0 (https://www.ibm.com/support/pages/spss-statistics-220-available-download).

Figure 7 shows regional activation in positive correlation with PMAT24_A_CR, with age, sex and years of education as covariates. Summarized in Table 3, these clusters involved biFPC, preSMA, dorsal precuneus, and rAI. Almost all of the clusters overlapped with those with activities in positive correlation with RT_2–0_ as highlighted in magenta in Fig. 5A. No clusters showed activation in negative correlation with PMAT24_A_CR. Because the correlation between PMAT24_A_CR and RT_2–0_ showed a very small effect size (Cohen’s f^2^ = 0.0246), we did not follow up with path or mediation analyses on these variables.

**Table 3:** Clusters showing correlation with PMAT24_A_CR, with age, sex, years of education as covariates.

Region	Cluster size (k)	Peak Voxel (T)	Cluster FWE P-value	MNI coordinate (mm)		
				X	Y	Z
**Positive**						
Parietal_Inf_R	90	6.50	0.000	44	− 46	44
Parietal_Inf_L	35	6.41	0.000	− 45	− 38	44
Supp_Motor_Area	302	6.37	0.000	− 2	10	48
Frontal_Mid_R	133	6.29	0.000	30	8	54
Parietal_Sup_L	61	5.86	0.000	− 28	− 58	48
Frontal_Mid_L	47	5.58	0.000	− 22	10	60
Precuneus	45	5.49	0.000	− 4	− 62	48
Insula_R	34	5.39	0.000	34	28	2

*R* right, *L* left.

To determine if the working memory-specific neural correlates engaged during the n-back task and predicting working memory performance also reflect Gf, we computed the beta values of the clusters and ran a regression of the beta value against PMAT performance scores (Gf). The results showed that the beta value of (CSI + RT −; dACC) was not correlated with Gf (r = − 0.013, p = 0.698, Cohen’s f^2^ = 0.000169). The beta value of (CSI − RT + ; preSMA, biFPC, and rAI) was only weakly correlated with Gf (r = 0.130, p = 0.000062, Cohen’s f^2^ = 0.01719).

## Discussion

Bilateral superior/middle/inferior frontal cortex, bilateral inferior parietal cortex, dorsomedial prefrontal cortex (in the area of the pre-SMA), bilateral caudate head, thalamus, and anterior insula showed higher activation during 2- vs. 0-back, in accord with earlier findings^14,36–44^. In the aims to characterize individual variation in behavioral performance and the neural correlates, we showed that, first, CSI_2–0_ was negatively correlated with the RT_2–0_, suggesting that “taking time to identify the target” did not improve the accuracy in 2- vs. 0-back; participants who performed with higher accuracy were also faster in identifying the target. The dACC showed activities during 2- vs. 0-back both in positive correlation with CSI_2–0_ and negative correlation with RT_2–0_. Further, path analyses showed a most significant fit of the model dACC → RT_2–0_ → CSI_2–0,_ suggesting a critical role of target switching and the dACC in determining performance accuracy. Individual variations in RT_2–0_ and Gf were positively correlated, though only with a small effect size, whereas CSI_2–0_ and Gf were not significantly correlated. We highlighted the main findings in discussion.

### Individual variation of CSI_2–0_ and RT_2–0_ in the N-back task

Individuals differ markedly in working memory performance, as reflected in the accuracy and RT (Supplementary Fig. S1). Here, the CSI_2–0_ was negatively correlated with the RT_2–0_, indicating that participants who were more accurate were also faster in identifying 2- vs. 0-back target. Conventionally, accuracy is emphasized without specific constraint on response time in the N-back task, which can lead to a ceiling or near-ceiling effect^13,45^. That is, individuals may intentionally slow down to optimize accuracy. However, we observed here that the CSI_2–0_ and RT_2–0_ were negatively correlated across subjects, suggesting that prolonging RT did not confer an advantage in achieving higher accuracy.

In whole-brain linear regressions we identified the correlates of individual variation in CSI_2–0_ and RT_2–0_. CSI_2–0_ was correlated with higher activation of a small cluster on the border of dACC and SMA and with lower activation of the preSMA, bilateral FPC and right AI. Despite a more limited coefficient of variation (CV = SD/mean; 0.61 for RT_2–0_ vs. 2.04 for CSI_2–0_), RT_2–0_ was associated with a wider swath of regional responses, in the preSMA, biFPC, thalamus, basal ganglia, dorsal precuneus, rAI, and cerebellum in positive correlation and in the dACC, middle and posterior cingulate, ventral precuneus, bilateral somatomotor cortex in negative correlation. This finding may suggest RT as a more sensitive index of regional responses to support working memory^13^ and individual variation reflecting not only differences in memory capacity but also the efficiency in utilizing the memory^46,47^. The preSMA showed higher activation in correlation with RT_2–0_, consistent with its role in decision making and controlled actions^48,49^. In contrast, somatomotor cortex showed higher activities in support of speedier responses during 2- vs. 0- back, consistent with earlier reports of motor cortical activities in relation to RT^50,51^.

It is possible that CSI_2–0_ may be determined with a number of different neural processes, including encoding, maintenance and target-updating during stimulus presentation, with each engaged to different degrees across subjects that altogether accounted for the individual variation in CSI_2–0_. In contrast, RT_2–0_ may more specifically reflect the process of target updating and identification, allowing its neural correlate to reveal in group regression. This contrast is reminiscent of earlier findings from the stop signal task^52–54^. Whereas individuals exhibited behavioral slowing following both stop success (SS) and stop error (SE) trials, a direct contrast between post-SE and post-go trials identified right hemispheric ventrolateral PFC activation but one between post-SS and post-go trials failed to demonstrate regional activities. We similarly argued that post-SS likely involved more complex mental processing, including motor hesitation, which differed too extensively across subjects to yield a consistent pattern of regional responses^53^.

### Neural processes inter-relating CSI_2–0_ and RT_2–0_

The dACC showed lower activation during 2- vs. 0- back blocks (Fig. 2), as also demonstrated in an earlier study^55^, seemingly in contrast with the role of the ACC in cognitive control. However, we observed across the whole-brain regressions dACC activity in positive correlation with CSI_2–0_ and negative correlation with RT_2–0_. Thus, less diminution of dACC activity during 2- vs. 0- back is associated with more efficient response and higher accuracy. These findings together suggest that while dACC is overall less engaged during 2- vs. 0- back, a greater extent of dACC engagement during 2-back would facilitate target identification. An extensive body of work demonstrated that the dACC responds to saliency and set switching^56–60^. For instance, in the monetary delay incentive task, pupil dilations were linked to increased activity in the dACC, which may trigger an increase in arousal to enhance task performance^61^. It is likely that the dACC was more engaged in 0- vs. 2- back because 0-back required simply target detection and focused, moment-to-moment attention whereas 2-back required attention to be distributed over the sequence of stimuli. On the other hand, higher dACC activity in switching the target identity would facilitate N-back performance. Notably, without clearly distinguishing the subregions, studies have reported higher activation of the medial prefrontal cortex to 2- vs. 0-back, but a closer examination revealed that the clusters, with Z coordinates ranging from + 40 to + 44, appeared to be largely in the preSMA^44,62^, as we also observed here.

A number of brain regions, including the preSMA, biFPC and rAI showed activation during 2- vs. 0-back in negative correlation with CSI_2–0_ and positive correlation with RT_2–0_. As described earlier, the preSMA is widely implicated in volitional, controlled action and decision making. For instance, the preSMA monitored conflict and facilitated slowing of motor response as a result of expected conflicts^49^. Bilateral FPC likewise is known for its role in restraining impulsive responses^63,64^, consistent with the current findings. While also considered as part of the salience network^61,65–68^, the AI showed higher activation during 2- vs. 0-back, suggesting that saliency alone cannot account for these regional activities. A recent study showed that the rAI increased in activity monotonically as a function of cognitive load in a backward masking majority function task^41^, broadly in accord with the current finding of higher response during 2- vs. 0-back. The AI has also been implicated in higher demand of effort across many other behavioral paradigms^69–71^. Together, these considerations highlight the multiple component processes involved in working memory; how areal activations are dedicated specifically to these component processes remain to be investigated.

Importantly, we showed in path analyses the model with the most significant fit: dACC activity → RT_2–0_ → CSI_2–0_, suggesting that, by way of enhancing target switching, the dACC decreases the RT and facilitates accuracy during 2- vs. 0- back. In contrast, the preSMA, biFPC and rAI did not significantly form paths with RT_2–0_ → CSI_2–0_. These findings support a central role of the dACC in supporting N-back performance, whereas the CSI − RT + clusters—preSMA, biFPC, and rAI—did not appear to partake specifically in relating RT_2–0_ to CSI_2–0_.

### Working memory and the Gf

Gf was not significantly correlated with CSI_2–0_ and only correlated with RT_2–0_ with a small effect size. Thus, Gf did not appear to be well reflected in individual N-back performance. On the other hand, both Gf and RT_2–0_ shared activations in positive correlation in the preSMA, bilateral but predominantly right FPC, rAI and the dorsal precuneus. These findings suggest that Gf was at best marginally captured by individual differences in RT_2–0_ and, to the extent these variances could be accounted for by regional brain activities, the activities reflect slower and perhaps more cautious responding during 2- vs. 0-back. While these results appear to be consistent with an earlier literature associating Gf with activities of the cognitive control network^72–74^ and with the parieto-frontal integration hypothesis of human intelligence^75–77^, a large proportion of the variance in Gf was notably not explained by RT_2–0_ or shared regional activities.

The same brain regions showed higher activations proportionally to the extent to which participants anticipated conflict and slowed down in the stop signal task^49,56,78^. This finding suggested that individuals with higher Gf were more inclined to strategize their RT and the preSMA, right FPC and AI support these behavioral processes. Post-conflict slowing reflects cognitive control. In the cognitive control model of human intelligence, cognitive control serves as a core component of working memory and intellectual abilities, especially the Gf, and drives the relationships between these constructs^14^. Regional activations in behavioral paradigms other than the N-back task may better capture individual differences in Gf.

### Limitations of the study and conclusions

A few limitations need to be considered. First, in the HCP N-back task, the stimulus was presented for 2 s, followed by an inter-trial interval of 0.5 s, in each trial, which allowed participants plenty of time (2.5 s in total) to respond to the target. However, the mean RTs were 989 and 790 ms for 2- and 0-back, respectively, suggesting that the majority of participants responded well before the trial ended most of the time. We reviewed 18 studies from the literature and noted that this appeared to be typically the case (Supplementary Table S3). Although we cannot speculate whether or how participants were engaged in the decision about speed-accuracy trade-off on the basis of these data, it is likely that regional brain activities were dictated by these task parameters and performance metrics, and the current findings should be considered as specific to the HCP. A study that systematically manipulates the task constraints within the same group of participants would be needed to thoroughly investigate how task parameters influence speed and accuracy and the neural processes underlying individual speed-accuracy trade-off in the N-back task. Second, although working memory is central to fluid intelligence, to what extent the N-back performance reflects Gf, as evaluated by the RSPM, represents a potential issue^6^ and suggests the need to consider the current findings on Gf as specific to RSPM. Third, the voxels that showed overlap between two sets of regressions were not identified on the basis of a statistical procedure. However, to our knowledge, there are no formal statistical approaches to assessing the significance of “overlap” between two sets of regressions (i.e., two different models). Conjunction or disjunction analyses, as implemented in SPM, could only be performed for different contrasts within the same GLM. Finally, although the HCP aimed to recruit “healthy populations,” the participants were heterogeneous in clinical characteristics, including many with history of or current substance use. Whereas we did not control for these variables, in the hope that, as true to the HCP, the data may reflect a broader population, the influences of the variables on the current findings remain to be clarified.

In conclusion, our findings highlight the key neural correlates of N-back performance metrics. The findings suggest the RT as a more sensitive measure of N-back performance and a key role of the dACC in supporting efficient target identification during 2- vs. 0-back.

## Supplementary Information

Supplementary Information.

## References

[1] Callicott JH (1999). Physiological characteristics of capacity constraints in working memory as revealed by functional MRI. Cereb. Cortex.

[2] Chatham CH (2011). From an executive network to executive control: A computational model of the n-back task. J. Cogn. Neurosci..

[3] Lamichhane B, Westbrook A, Cole MW, Braver TS (2020). Exploring brain-behavior relationships in the N-back task. Neuroimage.

[4] Yaple ZA, Stevens WD, Arsalidou M (2019). Meta-analyses of the n-back working memory task: fMRI evidence of age-related changes in prefrontal cortex involvement across the adult lifespan. Neuroimage.

[5] Jaeggi SM, Buschkuehl M, Perrig WJ, Meier B (2010). The concurrent validity of the N-back task as a working memory measure. Memory.

[6] Kane MJ, Conway ARA, Miura TK, Colflesh GJH (2007). Working memory, attention control, and the n-back task: A question of construct validity. J. Exp. Psychol. Learn. Mem. Cogn..

[7] Cole MW, Yarkoni T, Repovš G, Anticevic A, Braver TS (2012). Global connectivity of prefrontal cortex predicts cognitive control and intelligence. J. Neurosci..

[8] Nagel I (2011). Load modulation of BOLD response and connectivity predicts working memory performance in younger and older adults. J. Cogn. Neurosci..

[9] Gajewski PD, Hanisch E, Falkenstein M, Thönes S, Wascher E (2018). What does the n-back task measure as we get older? Relations between working-memory measures and other cognitive functions across the lifespan. Front. Psychol..

[10] Gilmour G (2019). Relating constructs of attention and working memory to social withdrawal in Alzheimer's disease and schizophrenia: Issues regarding paradigm selection. Neurosci. Biobehav. Rev..

[11] Israel M (2015). n-Back task performance and corresponding brain-activation patterns in women with restrictive and bulimic eating-disorder variants: Preliminary findings. Psychiatry Res. Neuroimag..

[12] Meule A (2017). Reporting and interpreting working memory performance in n-back tasks. Front. Psychol..

[13] Hur J, Iordan AD, Dolcos F, Berenbaum H (2017). Emotional influences on perception and working memory. Cogn. Emot..

[14] Chen Y (2019). Testing a cognitive control model of human intelligence. Sci. Rep..

[15] Engle RW, Tuholski SW, Laughlin JE, Conway A (1999). Working memory, short-term memory, and general fluid intelligence: A latent-variable approach. J. Exp. Psychol..

[16] Engle RW (2002). Working memory capacity as executive attention. Curr. Dir. Psychol. Sci..

[17] Nęcka E, Lulewicz A (2016). Capacity, control, or both: Which aspects of working memory contribute to children’s general fluid intelligence?. Pol. Psychol. Bull..

[18] Primi R (2001). Complexity of geometric inductive reasoning tasks: Contribution to the understanding of fluid intelligence. Intelligence.

[19] Jaeggi SM, Buschkuehl M, Jonides J, Perrig WJ (2008). Improving fluid intelligence with training on working memory. Proc. Natl. Acad. Sci..

[20] Lawlor-Savage L, Goghari VM (2016). Dual N-back working memory training in healthy adults: A randomized comparison to processing speed training. PLoS ONE.

[21] Burgess GC, Gray JR, Conway ARA, Braver TS (2011). Neural mechanisms of interference control underlie the relationship between fluid intelligence and working memory span. J. Exp. Psychol. Gen..

[22] Van Essen DC (2013). The WU-Minn human connectome project: An overview. Neuroimage.

[23] Bilker WB (2012). Development of abbreviated nine-item forms of the Raven’s standard progressive matrices test. Assessment.

[24] Wilks DS (2011). Statistical Methods in the Atmospheric Sciences.

[25] Kearney-Ramos TE (2014). Merging clinical neuropsychology and functional neuroimaging to evaluate the construct validity and neural network engagement of the n-back task. J. Int. Neuropsychol. Soc..

[26] Cohen J (1988). Statistical Power Analysis for the Behavioral Sciences.

[27] Van Essen DC (2012). The human connectome project: A data acquisition perspective. Neuroimage.

[28] Li G, Zhang S, Le TM, Tang X, Li C-SR (2020). Neural responses to reward in a gambling task: Sex differences and individual variation in reward-driven impulsivity. Cerebr. Cortex Commun..

[29] Li G, Zhang S, Le TM, Tang X, Li C-SR (2020). Neural responses to negative facial emotions: Sex differences in the correlates of individual anger and fear traits. Neuroimage.

[30] Kleber B (2016). Voxel-based morphometry in opera singers: Increased gray-matter volume in right somatosensory and auditory cortices. Neuroimage.

[31] Yan C-G, Wang X-D, Zuo X-N, Zang Y-F (2016). DPABI: Data processing & analysis for (resting-state) brain imaging. Neuroinformatics.

[32] Duvernoy HM (2009). The Human Brain.

[33] Chen F, Curran PJ, Bollen KA, Kirby J, Paxton P (2008). An empirical evaluation of the use of fixed cutoff points in RMSEA test statistic in structural equation models. Sociol. Methods Res..

[34] Hu L-T, Bentler PM (1995). Structural Equation Modeling: Concepts, Issues, and Applications.

[35] Le TM, Zhornitsky S, Zhang S, Li C-SR (2020). Pain and reward circuits antagonistically modulate alcohol expectancy to regulate drinking. Transl. Psychiatry.

[36] Barch DM, Sheline YI, Csernansky JG, Snyder AZ (2003). Working memory and prefrontal cortex dysfunction: Specificity to schizophrenia compared with major depression. Biol. Psychiat..

[37] Dehghan Nayyeri M, Burgmer M, Pfleiderer B (2019). Impact of pressure as a tactile stimulus on working memory in healthy participants. PLoS ONE.

[38] Li X (2019). Clinical utility of the dual n-back task in schizophrenia: A functional imaging approach. Psychiatry Res. Neuroimag..

[39] Fan J (2014). An information theory account of cognitive control. Front. Hum. Neurosci..

[40] Niendam TA (2012). Meta-analytic evidence for a superordinate cognitive control network subserving diverse executive functions. Cogn. Affect. Behav. Neurosci..

[41] Wu T (2019). Anterior insular cortex is a bottleneck of cognitive control. Neuroimage.

[42] Wu T (2020). The functional anatomy of cognitive control: A domain-general brain network for uncertainty processing. J. Comp. Neurol..

[43] Owen AM, McMillan KM, Laird AR, Bullmore E (2005). N-back working memory paradigm: A meta-analysis of normative functional neuroimaging studies. Hum. Brain Mapp..

[44] Ragland JD (2002). Working memory for complex figures: An fMRI comparison of letter and fractal n-back tasks. Neuropsychology.

[45] Hockey A, Geffen G (2004). The concurrent validity and test–retest reliability of a visuospatial working memory task. Intelligence.

[46] Luck SJ, Vogel EK (2013). Visual working memory capacity: From psychophysics and neurobiology to individual differences. Trends Cogn. Sci..

[47] Mackie M-A, Van Dam NT, Fan J (2013). Cognitive control and attentional functions. Brain Cogn..

[48] Hu S, Tseng Y-C, Winkler AD, Li C-SR (2014). Neural bases of individual variation in decision time. Hum. Brain Mapp..

[49] Hu S, Ide JS, Zhang S, Li C-SR (2015). Anticipating conflict: Neural correlates of a Bayesian belief and its motor consequence. Neuroimage.

[50] Hirose S, Nambu I, Naito E (2018). Cortical activation associated with motor preparation can be used to predict the freely chosen effector of an upcoming movement and reflects response time: An fMRI decoding study. Neuroimage.

[51] Mohamed MA, Yousem DM, Tekes A, Browner N, Calhoun VD (2004). Correlation between the amplitude of cortical activation and reaction time: A functional MRI study. Am. J. Roentgenol..

[52] Chang A, Chen C-C, Li H-H, Li C-SR (2014). Event-related potentials for post-error and post-conflict slowing. PLoS ONE.

[53] Li C-SR (2008). Neural correlates of post-error slowing during a stop signal task: A functional magnetic resonance imaging study. J. Cogn. Neurosci..

[54] Zhang Y (2017). Distinct neural processes support post-success and post-error slowing in the stop signal task. Neuroscience.

[55] Gray JR, Braver TS (2002). Personality predicts working-memory—related activation in the caudal anterior cingulate cortex. Cogn. Affect. Behav. Neurosci..

[56] Ide JS, Shenoy P, Yu AJ, Li C-SR (2013). Bayesian prediction and evaluation in the anterior cingulate cortex. J. Neurosci..

[57] Li S (2017). Novelty seeking and reward dependence-related large-scale brain networks functional connectivity variation during salience expectancy. Hum. Brain Mapp..

[58] Manza P (2016). A dual but asymmetric role of the dorsal anterior cingulate cortex in response inhibition and switching from a non-salient to salient action. Neuroimage.

[59] Zhang Y (2019). Structural connectivity profile supports laterality of the salience network. Hum. Brain Mapp..

[60] Manza P (2016). The effects of methylphenidate on cerebral responses to conflict anticipation and unsigned prediction error in a stop-signal task. J. Psychopharmacol..

[61] Schneider M, Leuchs L, Czisch M, Sämann PG, Spoormaker VI (2018). Disentangling reward anticipation with simultaneous pupillometry/fMRI. Neuroimage.

[62] Carlson S (1998). Distribution of cortical activation during visuospatial n-back tasks as revealed by functional magnetic resonance imaging. Cereb. Cortex.

[63] Cai W, Chen T, Ide JS, Li C-SR, Menon V (2017). Dissociable fronto-operculum-insula control signals for anticipation and detection of inhibitory sensory cue. Cereb. Cortex..

[64] Hu S, Ide JS, Zhang S, Li C-SR (2016). The right superior frontal gyrus and individual variation in proactive control of impulsive response. J. Neurosci..

[65] Farr OM, Hu S, Zhang S, Li C-SR (2012). Decreased saliency processing as a neural measure of Barratt impulsivity in healthy adults. Neuroimage.

[66] Kann S, Zhang S, Manza P, Leung H-C, Li C-SR (2016). Hemispheric lateralization of resting-state functional connectivity of the anterior insula: Association with age, gender, and a novelty-seeking trait. Brain. Connect..

[67] Le TM, Zhang S, Zhornitsky S, Wang W, Li C-SR (2020). Neural correlates of reward-directed action and inhibition of action. Cortex.

[68] Zhang J-T (2016). Altered resting-state functional connectivity of the insula in young adults with Internet gaming disorder. Addict. Biol..

[69] Aben B, BucCalderon C, Van den Bussche E, Verguts T (2020). Cognitive effort modulates connectivity between dorsal anterior cingulate cortex and task-relevant cortical areas. J. Neurosci..

[70] Chong TTJ (2017). Neurocomputational mechanisms underlying subjective valuation of effort costs. PLoS Biol..

[71] Müller T, Apps MAJ (2019). Motivational fatigue: A neurocognitive framework for the impact of effortful exertion on subsequent motivation. Neuropsychologia.

[72] Colom R (2009). Gray matter correlates of fluid, crystallized, and spatial intelligence: Testing the P-FIT model. Intelligence.

[73] Preusse F, van der Meer E, Deshpande G, Krueger F, Wartenburger I (2011). Fluid intelligence allows flexible recruitment of the parieto-frontal network in analogical reasoning. Front. Hum. Neurosci..

[74] Peters M (1995). A Redrawn Vandenberg and Kuse mental rotations test: Different versions and factors that affect performance. Brain Cogn..

[75] Colom R, Karama S, Jung RE, Haier RJ (2010). Human intelligence and brain networks. Dialog. Clin. Neurosci..

[76] Jung RE, Haier RJ (2007). The Parieto-Frontal Integration Theory (P-FIT) of intelligence: Converging neuroimaging evidence. Behav. Brain Sci..

[77] Vakhtin AA, Ryman SG, Flores RA, Jung RE (2014). Functional brain networks contributing to the Parieto-Frontal integration theory of intelligence. Neuroimage.

[78] Wang W (2018). Motor preparation disrupts proactive control in the stop signal task. Front. Hum. Neurosci..

