# Supplementary material for: Neural correlates of individual variation in two-back working memory and the relationship with fluid intelligence
Source: Sci Rep. 2021 May 11;11:9980. doi: 10.1038/s41598-021-89433-8 (PMC8113462; doi:10.1038/s41598-021-89433-8)
Supplement: Supplementary file 1 — Supplementary Information. [file 41598_2021_89433_MOESM1_ESM.docx]

**Supplement**

**Li et al.**

**Neural correlates of individual variation in two-back working memory and the relationship with fluid intelligence**

Guangfei Li ^1,2,^^, Yu Chen ^2,^^, Thang M. Le ^2^, Wuyi Wang ^2^, Xiaoying Tang ^1,*^, Chiang-Shan R. Li ^2,3,4,*^

^1^Department of Biomedical engineering, School of Life Sciences, Beijing Institute

of technology, Beijing, China

^2^Department of Psychiatry, Yale University School of Medicine, New Haven, CT, USA

^3^Department of Neuroscience, Yale University School of Medicine, New Haven,

CT, USA

^4^Interdepartmental Neuroscience Program, Yale University School of Medicine,

New Haven, CT, USA

**Supplementary Table S1.** Regional activations to 2- vs. 0- back: one-sample T-test; voxel p<0.05, FWE corrected.

| Region | Cluster size (k) | Peak Voxel (T) | Cluster FWE  P- value | MNI coordinate (mm) | | |
| --- | --- | --- | --- | --- | --- | --- |
|  |  |  |  | X Y Z | | |
| *2- > 0- back* |  |  |  |  |  |  |
| Frontal_Sup_R | 14521 | 33.41 | 0.000 | 24 | 6 | 58 |
| Parietal_Inf_R | 3993 | 31.52 | 0.000 | 42 | -44 | 48 |
| Pulvinar* | 316 | 12.46 | 0.000 | 2 | -28 | -2 |
| White matter* | 116 | 10.40 | 0.000 | -2 | 12 | 24 |
| Cerebellum | 67 | 8.29 | 0.000 | -2 | -44 | -16 |
| White matter* | 74 | 7.61 | 0.000 | -18 | -38 | 14 |
|  |  |  |  |  |  |  |
| *2- < 0- back* |  |  |  |  |  |  |
| Cingulum_Post_L | 14941 | -27.24 | 0.000 | -2 | -48 | 30 |
| Frontal_Sup_L | 343 | -21.74 | 0.000 | -12 | 52 | 36 |
| Frontal_Sup_Medial_R | 114 | -11.85 | 0.000 | 8 | 52 | 38 |
| Thalamus_R | 44 | -8.34 | 0.000 | 0 | -14 | 6 |

**Supplementary Table S2**. Statistics of path analyses of CSI_2-0_, RT_2-0_ and brain activation, with age, sex, years of education as covariates.

|  | CFI | RMSEA | SRMR | Chi square/df |
| --- | --- | --- | --- | --- |
| Models of X (CSI_2-0_), Y (RT_2-0_) and Z (CSI_2-0_+RT_2-0_-) | | | | |
| X→Y→Z | 0.981 | 0.077 | 0.016 | 6.663 |
| X→Z→Y | 0.653 | 0.329 | 0.065 | 103.548 |
| Y→X→Z | 0.842 | 0.222 | 0.043 | 47.663 |
| Y→Z→X | 0.653 | 0.329 | 0.065 | 103.548 |
| Z→X→Y | 0.842 | 0.222 | 0.043 | 47.663 |
| **Z→Y→X** | **0.981** | **0.077** | **0.016** | **6.663** |
| Models of X (CSI_2-0_), Y (RT_2-0_) and Z (CSI_2-0_-RT_2-0_+) | | | | |
| X→Y→Z | 0.923 | 0.182 | 0.034 | 32.424 |
| X→Z→Y | 0.825 | 0.274 | 0.051 | 72.315 |
| Y→X→Z | 0.772 | 0.314 | 0.058 | 94.365 |
| Y→Z→X | 0.825 | 0.274 | 0.051 | 72.315 |
| Z→X→Y | 0.772 | 0.314 | 0.058 | 94.365 |
| Z→Y→X | 0.923 | 0.182 | 0.034 | 32.424 |

**Supplementary Table S3.** N-back studies with data on RT and accuracy

|  | | Mean age, years | Stimulus duration, ms | Inter-trial-interval, ms | Time window, s | Mean RT, ms | | Mean accuracy, % | |
| --- | --- | --- | --- | --- | --- | --- | --- | --- | --- |
|  |  |  |  |  |  | 0-back | 2-back | 0-back | 2-back |
| ^1^ | 21.3 | | 500 | 1500 | / | 405 | 441 | NA* | NA* |
| ^2^ | 25.5 | | 1300 | 200 | / | 510 | 740 | 98 | 94 |
| ^3^ | 29.0 | | 500 | 2000 | / | 634 | 1065.5 | 99.1 | 80.9 |
| ^4^ | 31.3 | | 3000 | 0 | / | 785 | 467 | 98 | 94.5 |
| ^5^ | 29.8 | | 500 | 1000 | / | 490 | 547 | 95.7 | 88.9 |
| ^6^ | 24.8 | | 500 | 1500 | / | 5284 | 6052 | 99 | 95 |
| ^7^ | 24.1 | | 500 | 2000/2500 | / | / | / | 90.63 | 72.92 |
| ^8^ | 20.5 | | / | / | 5 | 2000 | 1930 | 83.28 | 61.66 |
| ^9^ | 23.4 | | 500 | 1500 | / | 430 | 459 | 97.62 | 94.05 |
| ^10^ | 37.9 | | 500 | 1000 | / | / | / | 95 | 93 |
| ^11^ | 23.8 | | 500 | 2000 | / | 647 | 866 | 95.14 | 92.36 |
| ^12^ | 32.2 | | 500 | 2500 | / | 520 | 635 | 95 | 83 |
| ^13^ | 32.0 | | 1200 | 300 | / | 451 | 632 | 97 | 61 |
| ^14^ | 22.9 | | 1700 | 300 | / | 556.48 | 662.21 | 99.6 | 98.7 |
| ^15^ | 24.4 | | 200 | 800 | / | 462 | 369 | 84.1 | 65.3 |
| ^16^ | 35.8 | | 1000 | 500 | / | 536.2 | 714.1 | 88.7 | 80.1 |
| ^17^ | 34.4 | | 2000 | / | / | 545 | 650 | 100 | 90.20 |
| ^18^ | 30.0 | | 500 | 2500 | / | 682 | 806 | 96 | 92 |

Note: 1. If both patients and healthy controls were included in the study, we showed here only the data of healthy controls; 2. If several different N-back tasks were involved in the study, we showed here the data collected of the task most similar to HCP N-back task; 3. For some studies where the RT and/or AR were shown in plots (e.g., bar graphs) without numerical data, we estimated the RT and/or AR based on the plots. Time window: the time within which the subjects were to make a response – responses beyond the time window were considered as an error. “/”: no data. *Data were replaced by other measures; in [1], accuracy was recorded using d’, which accounted for correct responses and false alarms: d’ = *Z*(*hit rate*) − *Z*(*false alarm rate*). In [7], ITI=2000/2500; 2000 for 0-back and 2500 for 2-back.


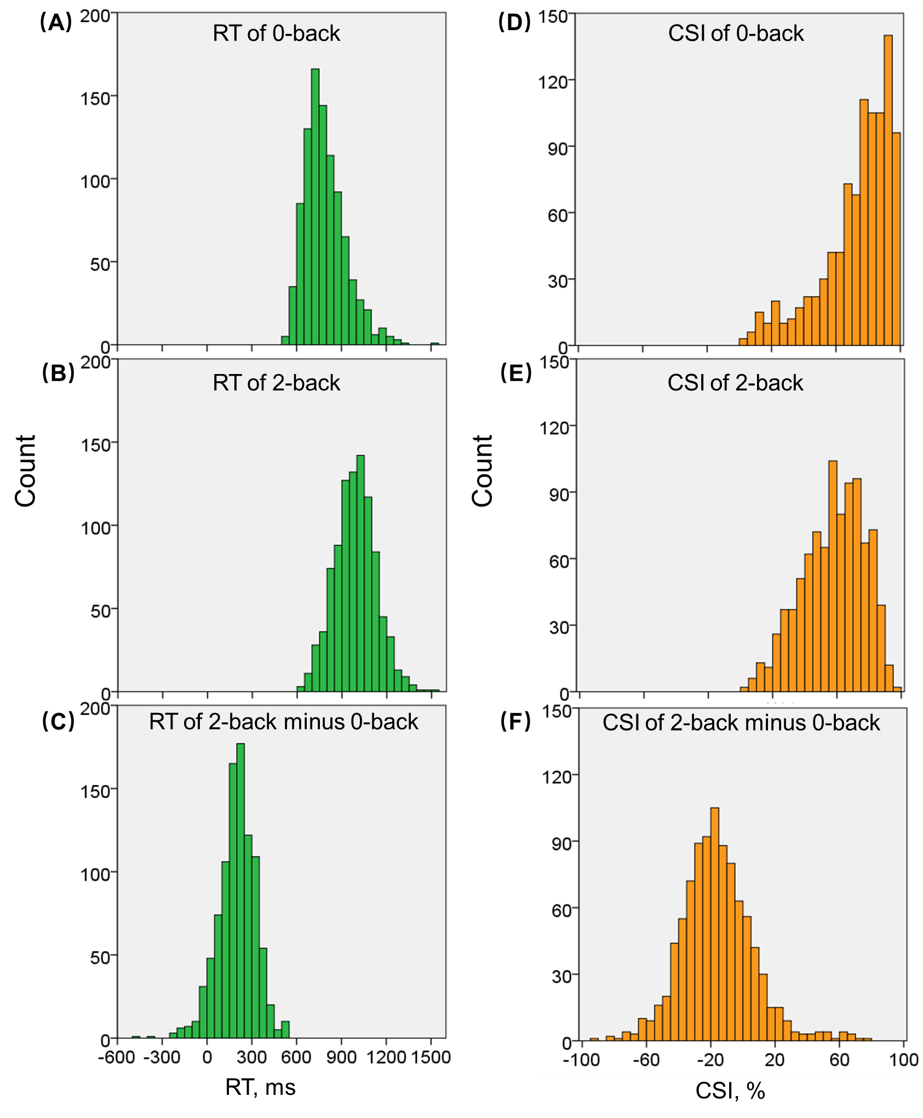


**Supplementary Figure S1.** Distribution of RT and CSI in 0-back and 2-back blocks and difference in RT and CSI between 0- and 2-back blocks. **Figure S1** was generated by SPSS Statistics 22.0 (https://www.ibm.com/support/pages/spss-statistics-220-available-download).


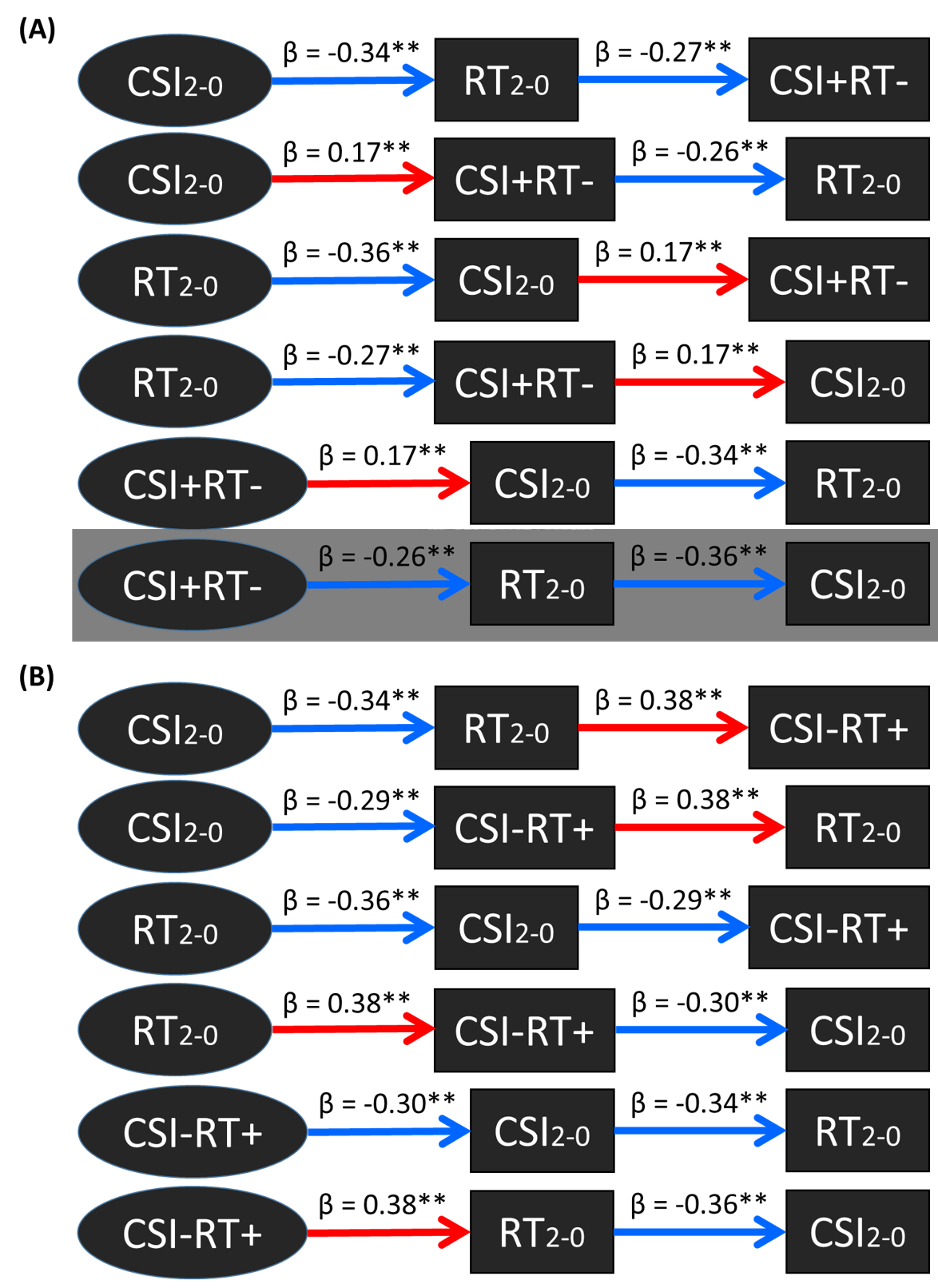


**Supplementary Figure S2.** Path analyses between **(A)** brain activation of cluster CSI_2-0_+RT_2-0_-, CSI_2-0_ and RT_2-0_-; and between **(B)** brain activation of cluster CSI_2-0_-RT_2-0_+, CSI_2-0_ and RT_2-0_-. The model with a significant fit was highlighted by gray background. Red arrows and blue arrows indicate positive and negative relationship, respectively. ***p* < 0.001.

**Supplementary References**

1 Wu, T. *et al.* The functional anatomy of cognitive control: A domain-general brain network for uncertainty processing. *Journal of Comparative Neurology* **528**, 1265-1292, doi:10.1002/cne.24804 (2020).

2 Thürling, M. *et al.* Involvement of the cerebellar cortex and nuclei in verbal and visuospatial working memory: A 7T fMRI study. *NeuroImage* **62**, 1537-1550, doi:<https://doi.org/10.1016/j.neuroimage.2012.05.037> (2012).

3 Harvey, P. O. *et al.* Cognitive control and brain resources in major depression: an fMRI study using the n-back task. *Neuroimage* **26**, 860-869, doi:10.1016/j.neuroimage.2005.02.048 (2005).

4 Rose, E. J., Simonotto, E. & Ebmeier, K. P. Limbic over-activity in depression during preserved performance on the n-back task. *NeuroImage* **29**, 203-215, doi:<https://doi.org/10.1016/j.neuroimage.2005.07.002> (2006).

5 Koshino, H. *et al.* Functional connectivity in an fMRI working memory task in high-functioning autism. *NeuroImage* **24**, 810-821, doi:<https://doi.org/10.1016/j.neuroimage.2004.09.028> (2005).

6 Jacola, L. M. *et al.* Clinical utility of the N-back task in functional neuroimaging studies of working memory. *Journal of clinical and experimental neuropsychology* **36**, 875-886, doi:10.1080/13803395.2014.953039 (2014).

7 Li, X. *et al.* Clinical utility of the dual n-back task in schizophrenia: A functional imaging approach. *Psychiatry Research: Neuroimaging* **284**, 37-44, doi:<https://doi.org/10.1016/j.pscychresns.2019.01.002> (2019).

8 Israel, M. *et al.* n-Back task performance and corresponding brain-activation patterns in women with restrictive and bulimic eating-disorder variants: Preliminary findings. *Psychiatry Research: Neuroimaging* **232**, 84-91, doi:<https://doi.org/10.1016/j.pscychresns.2015.01.022> (2015).

9 Livny, A. *et al.* The effects of synthetic cannabinoids (SCs) on brain structure and function. *European Neuropsychopharmacology* **28**, 1047-1057, doi:<https://doi.org/10.1016/j.euroneuro.2018.07.095> (2018).

10 Flanagan, J. C. *et al.* Effects of oxytocin on working memory and executive control system connectivity in posttraumatic stress disorder. *Exp Clin Psychopharmacol* **26**, 391-402, doi:10.1037/pha0000197 (2018).

11 Dehghan Nayyeri, M., Burgmer, M. & Pfleiderer, B. Impact of pressure as a tactile stimulus on working memory in healthy participants. *PloS one* **14**, e0213070-e0213070, doi:10.1371/journal.pone.0213070 (2019).

12 Ragland, J. D. *et al.* Working memory for complex figures: an fMRI comparison of letter and fractal n-back tasks. *Neuropsychology* **16**, 370-379 (2002).

13 Kearney-Ramos, T. E. *et al.* Merging clinical neuropsychology and functional neuroimaging to evaluate the construct validity and neural network engagement of the n-back task. *J Int Neuropsychol Soc* **20**, 736-750, doi:10.1017/S135561771400054X (2014).

14 Jung, K. *et al.* Effective connectivity during working memory and resting states: A DCM study. *NeuroImage* **169**, 485-495, doi:<https://doi.org/10.1016/j.neuroimage.2017.12.067> (2018).

15 Blokland, G. A. M. *et al.* Quantifying the heritability of task-related brain activation and performance during the N-back working memory task: a twin fMRI study. *Biol Psychol* **79**, 70-79, doi:10.1016/j.biopsycho.2008.03.006 (2008).

16 Yüksel, D. *et al.* Neural correlates of working memory in first episode and recurrent depression: An fMRI study. *Progress in Neuro-Psychopharmacology and Biological Psychiatry* **84**, 39-49, doi:<https://doi.org/10.1016/j.pnpbp.2018.02.003> (2018).

17 Schmidt, H. *et al.* No gender differences in brain activation during the N-back task: an fMRI study in healthy individuals. *Human brain mapping* **30**, 3609-3615, doi:10.1002/hbm.20783 (2009).

18 Philip, N. S. *et al.* Exposure to childhood trauma is associated with altered n-back activation and performance in healthy adults: implications for a commonly used working memory task. *Brain Imaging Behav* **10**, 124-135, doi:10.1007/s11682-015-9373-9 (2016).
